# A160 MOLECULAR ANALYSIS OF THE INJURY-REPAIR RESPONSE IN ULCERATIVE COLITIS REVEALS HETEROGENEITY IN DISEASE ACTIVIT

**DOI:** 10.1093/jcag/gwab049.159

**Published:** 2022-02-21

**Authors:** K Madill-Thomsen, J Venner, S Pon, K Kroeker, F Peerani, L A Dieleman, K Wong, D C Baugmart, P Halloran, B Halloran

**Affiliations:** 1 University of Alberta Faculty of Medicine & Dentistry, Edmonton, AB, Canada; 2 University of Manitoba, Winnipeg, MB, Canada; 3 Medicine, University of Alberta, Edmonton, AB, Canada

## Abstract

**Background:**

Ulcerative colitis (UC) is a chronic inflammatory condition affecting the colonic epithelium. We used an established microarray-based system to analyze a set of 128 UC biopsies (113 patients), assessing gene expression associated with the colon’s response to injury in UC.

**Aims:**

Our aim was to describe the burden of injury in UC biopsies and to explore molecular heterogeneity across disease activity, as assessed by the endoscopic Mayo score.

**Methods:**

128 UC colon biopsies were collected at the University of Alberta Hospital (Edmonton, AB) and Cedars-Sinai Hospital (Los Angeles, CA) during standard of care colonoscopy and processed using Affymetrix microarrays. Principal component analysis (PCA) and archetypal analysis (AA) visualized relationships between biopsies and previously annotated injury-associated transcript sets. AA assigned each biopsy to one of three groups, and scores to each biopsy relating it to all three groups.

**Results:**

Spearman correlations (**Table 1A**) were highest between the endoscopic Mayo score and the injury-repair-associated transcripts (IRRAT, 0.64, *P*=4.7x10^-16^), immunoglobulin transcripts highly associated with chronic injury and fibrosis (IGT, 0.63, P=3.0x10^-15^), endothelial transcripts (ENDAT, 0.61, *P*=1.8x10^-14^), and parenchymal dedifferentiation i.e. epithelial solute carrier loss (CT2, -0.60, *P*=6.5x10^-14^).

PCA separated injury from no injury in PC1 (**Figure 1A**). T cell transcripts (QCATs), interferon-gamma inducible transcripts (GRITs) and targets of biologics (IL12, TNFA, ITGA4/B7) separated from injury transcripts in PC2.

We assigned three AA groups and visualized biopsies in PCA (**Figure 1B**, colored by AA membership). Group 1 (grey, N=44) biopsies had little parenchymal dedifferentiation and low expression of injury-associated transcripts. Groups 2 (red, N=44) and 3 (blue, N=40) had increased expression of injury-associated transcript sets and dedifferentiation compared to Group 1 (**Table 1**). Although Group 3 was endoscopically similar to Group 1 (P>0.05), Group 3 showed elevated injury-associated transcript set expression (e.g. IRRAT) and increased parenchymal dedifferentiation (CT2).

**Conclusions:**

Assessment of UC biopsies using AA and previously annotated injury-associated gene sets reveals two groups of biopsies that are endoscopically similar though one group has increased molecular abnormalities, thus revealing heterogeneity unrelated to the Mayo score. A molecular system based around PCA and AA could enhance and refine UC disease assessment by allowing for quantitation and qualification of injury in biopsies obtained at endoscopy i.e. a level of resolution beyond conventional endoscopic scoring.

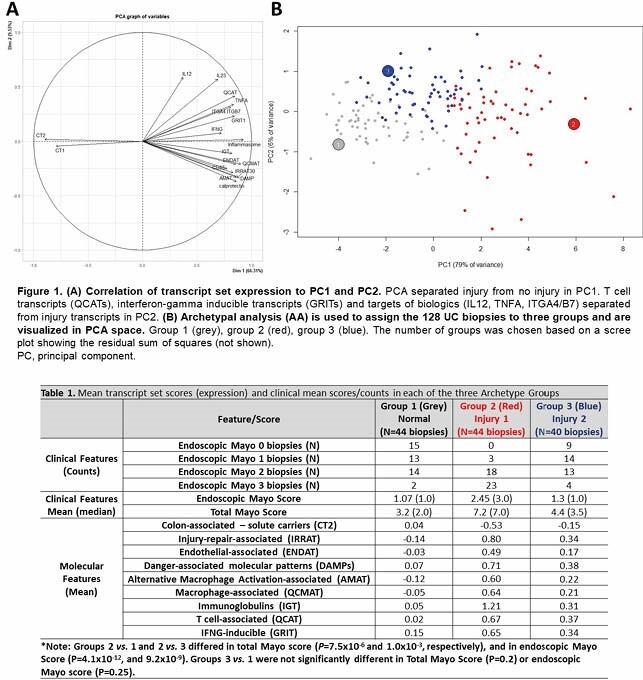

**Funding Agencies:**

None

